# Delayed chylothorax in the absence of radiological evidence of rib or vertebral body fractures following blunt trauma

**DOI:** 10.1093/jscr/rjab112

**Published:** 2021-04-06

**Authors:** Khurum Mazhar, Saifullah Mohamed, Akshay Jatin Patel, Sarah Berger-Veith, Qamar Abid, Shilajit Ghosh

**Affiliations:** Department of Thoracic Surgery, Royal Stoke University Hospital, Stoke-on-Trent ST4 6QG, UK; Department of Thoracic Surgery, Royal Stoke University Hospital, Stoke-on-Trent ST4 6QG, UK; Institute of Immunology and Immunotherapy, University of Birmingham, Birmingham B15 2TT, UK; Faculty of Medicine Carl Gustav Carus, TU Dresden, Dresden, Germany; Department of Thoracic Surgery, Royal Stoke University Hospital, Stoke-on-Trent ST4 6QG, UK; Department of Thoracic Surgery, Royal Stoke University Hospital, Stoke-on-Trent ST4 6QG, UK

**Keywords:** chylothorax, trauma, injury, chest, thoracic duct, medium chain triglyceride (MCT)

## Abstract

Our case report illustrates effective implementation of conservative measures without the need for more invasive procedures, which can be required in refractory cases. Our patient was a 42-year-old female who fell from a horse and presented with a 1-week history of dyspnoea. Investigations revealed her to have a large right chylothorax, which was treated conservatively with chest drainage and octreotide. The patient remained in hospital for a total of 3 days prior to being discharged home without further complications. Blunt traumatic chylothorax should be considered as part of the differential diagnosis in patients who present with ongoing dyspnoea or chest discomfort within a 2-week preceding history of blunt trauma. Radiological imaging should be mandatory and the absence of posterior thoracic fractures does not exclude the diagnosis. Conservative management with pleural drainage, medium-chain triglyceride diet and octreotide yielded excellent results in our case.

## INTRODUCTION

Blunt traumatic chylothorax (BTC) is a rare sequelae of thoracic trauma accounting between 0.2 and 1% of cases [1]. Unlike direct penetrating or iatrogenic injury, blunt traumatic rupture of the thoracic duct (TD) has three proposed mechanisms: (i) hyperextension/flexion of the thoracic spine, (ii) shear stress of the right diaphragmatic crus and (iii) posterior fracture of ribs/vertebrae [2]. However, there are limited number of case reports and series in the literature. We have summarized the findings of 24 documented cases (excluding ours) in the English language in the past 30 years in Table 1.

**Table 1:** Showing suspected cause of BTC and laterality of pathology in 24 case reports/series via ‘Pubmed MEDLINE’ literature search between 1990 and 2020 (available as full text in the English language). Categories are reported where documented by the original author(s)

Aetiology	Number of cases (n)
RTC	15
Fall	5
Fracture/dislocation site	
Ribs only	5
Vertebrae only	1
Both	7
None	2
Laterality of chylothorax	
Right	11
Left	8
Bilateral	5
Treatment with thoracotomy and TD ligation	6
Conservative management	18

RTC, road traffic collision.

## CASE PRESENTATION

**Figure 1:**
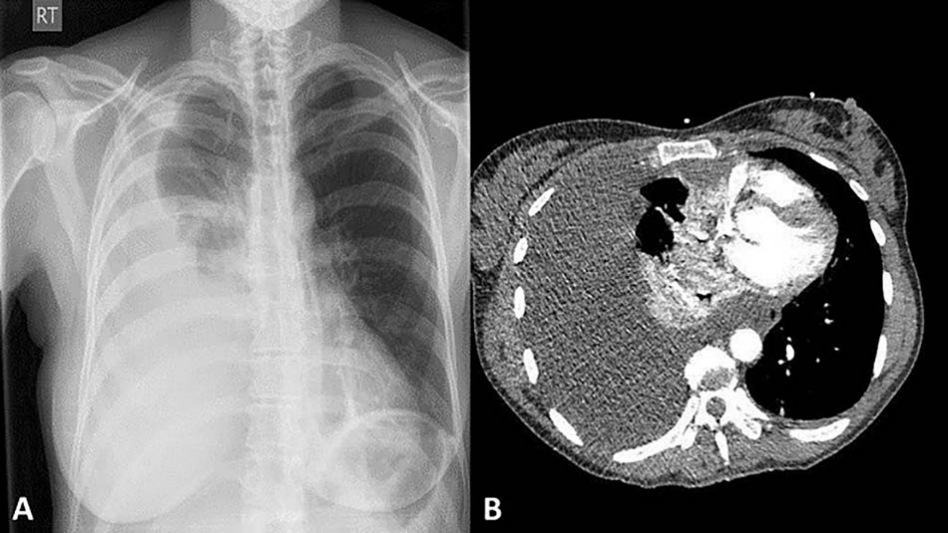
A postero-anterior plain CXR showing a large right-sided pleural effusion (**A**); a contrast-enhanced CT scan showing large right-sided pleural effusion causing right lung collapse and left mediastinal shift with compression of the right atrium; note the intact ribs and vertebra (**B**).

A 42-year-old female presented to the Accident and Emergency department with worsening dyspnoea. This was precipitated by a 6 ft fall from a bucked horse 1 week prior to presentation, in which, she sustained injuries to her back. Her vital observations were within the normal range and the pulse oximetry oxygen saturation was 100% on room air. There were decreased breath sounds on the auscultation of her right lung base and some minor bruising noted over the mid-thoracic spine area. A plain chest X-ray (CXR) (Fig. 1A) showed a right sided pleural effusion. A contrast-enhanced computed tomography (CT) scan of her thorax (Fig. 1B) revealed a large effusion with low Hounsfield Units and consistent with non-haemorrhagic material. There was a noted absence of rib fractures, but it did show a single non-displaced fracture of the T10 spinous process. Vigilance was maintained given the difficulty in detecting even minor bony injuries on cross-sectional imaging allied with the mechanism of injury.

A 32 French thoracostomy drain was used to drain 1800 ml of chylous appearing fluid. Biochemical testing of the fluid showed elevated triglyceride levels >1000 mg/dl.

The patient was treated with intravenous octreotide (somatostatin analogue) and a medium-chain triglyceride (MCT) diet. Another 1880 ml was drained in the next 24 h, but this reduced to just 40 ml on the third day after admission. The chest drain was removed and the patient was monitored for another 24 h prior to being discharged to her home after a plain CXR, confirming the resolution of the effusion.

## DISCUSSION

Two groups have reported chylothorax in the absence of fracture/dislocation of ribs/vertebra [3, 4]. Four cases did not give a cause of BTC, and it is noteworthy that not all reported cases were investigated with CT scanning, probably due to the variation of availability over the study period, including centres in the developing world. Almost half of the cases we found were right-sided BTC, perhaps owing to the TD’s longer course on this side and susceptibility to injury at the crus. Approximately, one-fifth of patients had bilateral BTC. Seventy-five percent of reported cases had successful treatment with conservative means.

Patients typically present with worsening dyspnoea and chest discomfort rather than pleurisy. Left unabated, it can cause hypovolaemia, respiratory distress, malnourishment, immunosuppression and mediastinal tension. Symptoms typically develop 1–7 days later, yet latency periods of years have been reported [5].

A preceding history of blunt trauma should be sought. Drainage of a milky white fluid from the thorax will exclude haemothorax or transudative effusion from diagnosis. Differential diagnoses are listed in Table 2, but concomitant injuries of the posterior ribs and/or vertebral fractures may be indicative of chylothorax. High triglyceride level of the effusion on biochemical analysis, >110 mg/dl, will confirm the diagnosis.

**Table 2:** Showing differential diagnosis of ‘white/milky’ pleural fluid

	Chylothorax	Pseudochylothorax	Empyema	Extravasation of TPN via subclavian line
Definition	Chyle within pleural cavity	High cholesterol content in pleural fluid	Pus in the pleural space	TPN in pleural fluid
Onset	Acute	Chronic	Acute	Acute
Odour	Odourless	Odourless	Repugnant	Odourless
Centrifugation appearance	Uniform	Uniform	Clear supernatant	Uniform
TG content	>110 mg/dl	>1–<50 mg/dl	Negative	>110 mg/dl
Addition of ethyl ether	No change	Clears fluid	Little effect	No change
Additional tests	Low potassium and glucose content			High potassium and glucose content

TG, triglyceride; TPN, total parenteral nutrition.

The objective of treatment is to (i) facilitate lung re-expansion by drainage of the pleural space, (ii) prevent malnutrition and (iii) decrease chyle production. Consensus favours the initial conservative management with chest drainage and a diet very low in long chain fats and supplemented with MCT diet. MCTs are absorbed directly into the portal circulation and hence bypass the lymphatic system. Many centres [6] favour use of octreotide to decrease chylous effusions. The conservative approach has been reported to have almost 90% success rate of all traumatic chylothoraces (including iatrogenic). In our review of the literature, we found 6 out of 24 cases who had successful (not necessarily warranted) thoracotomy and open ductal ligation for BTC. The others were successfully treated with a conservative approach. Most authors agree that failure of conservative measures after 2 weeks or drainage greater than 200 ml for 1–3 weeks necessitates a more invasive approach. The advantage of earlier surgical intervention includes a decrease in length of hospital stay and potential complications of persistent chylothorax, but the timing of this aggressive intervention remains controversial. Failure of prolonged conservative management risks malnourishment, and patients may require to be nil by mouth and receive total parenteral nutrition (TPN).

Ligation of the TD is reported to have a 90% success for all-cause chylothorax if performed above the right hemi-diaphragm [2], and chyle systemic return is facilitated by collateral circulation. Administration of methylene blue to olive oil consumption can be used to help identify the site of leak using lymphangiography. Lymphangiography did not form part of our investigation panel, but in the event of failure of the effusion to resolve with conservative measures, this would have been an appropriate next step in order to identify the site of TD injury and hence plan for further management steps, such as potential duct ligation. Where this remains difficult, pleural symphysis techniques may be beneficial. Patients unfit for major surgery can also have a pleuroperitoneal shunt minimizing the complications seen from chylothorax. Furthermore, certain groups [7, 8] have reported on the use of medical pleurodesis to manage refractory chylothoraces with the view that chylous fistulas are thought to close by obliteration of the adjacent pleural space as opposed to healing of the lymphatic vessels themselves. The use of bleomycin [7] and talc [8] have been reported to be effective and feasible in this group of patients, with success rates of 100% reported with talc at a 90-day follow-up [8].

Both Pamarthi *et al*. [9] and Itkin *et al*. [10], using TD embolization, report successful resolution of traumatic chylothorax in 79 and 71% of patients in 105 and 109 cases, respectively. Both advocate this as a safe, feasible and effective option in the initial treatment of chylothorax.

## CONFLICT OF INTEREST STATEMENT

None declared.

## FUNDING

None.
